# Participatory systems modelling for youth mental health: agility and adaptiveness to enhance stakeholder engagement and knowledge sharing

**DOI:** 10.1186/s13033-025-00687-5

**Published:** 2025-10-24

**Authors:** Sarah Piper, Victoria Loblay, Yun Ju Christine Song, Grace Yeeun Lee, Samantha Huntley, Olivia Iannelli, Nicholas Ho, Seyed Hossein Hosseini, Catherine Vacher, Alexis Hutcheon, Paul Crosland, Kristen Tran, Kim-Huong Nguyen, Chloe Gosling, Jordan van Rosmalen, Kayla Andrade, Ian B. Hickie, Jo-An Occhipinti

**Affiliations:** Brain and Mind Centre, Faculty of Medicine and Health, Translational Research Collective, The University of Sydney, Sydney, Australia

**Keywords:** Youth mental health, Participatory systems modelling, Stakeholder engagement, Knowledge sharing, Decision making, Diversity, Representation

## Abstract

**Background:**

Australia’s mental health system needs to expand rapidly to meet the growing demand for care by young Australians. Participatory systems modelling (PSM) has emerged as a valuable method for guiding strategic decision-making in mental health policy.

**Methods:**

This paper evaluates the participatory methods and approaches utilised in a series of PSM workshops focused on the development of a youth mental health decision-support tool for the Brisbane South region, Queensland. Baseline and two follow-up timepoints of semi-structured interviews were conducted with a range of local stakeholders, including mental health professionals, service managers, commissioning organisations, and young people with lived experience.

**Results:**

Participants emphasised the need for diversity of stakeholder representation in workshops, but acknowledged the challenge of recruiting young stakeholders and culturally diverse stakeholders. Clear communication and education around the decision-support tool, as well as the utilisation of flexible methods for obtaining stakeholder input, both served to empower stakeholders in their contributions to the workshops and strengthen stakeholder engagement and knowledge sharing.

**Conclusions:**

The adoption of more adaptive and flexible workshop activities, and a move away from more structured systems modelling workshop ‘scripts’, is required to engage diverse participants within the youth mental health space. Results suggest knowledge sharing and stakeholder engagement is an active process that is developed along the course of the workshops, enabled by education and clear communication, empowering participants to meaningfully contribute. Future PSM workshops should continue to develop additional activities and more targeted engagement with youth stakeholders to enhance their contributions.

## Introduction

Nearly one billion people worldwide were living with a mental Health disorder in 2019, including 14% of the world’s adolescents [65]. Governments across the globe face sustained pressure to address this crisis and invest in solutions, whilst navigating significant economic, environmental, and social challenges (The Lancet Public [55]). Australia is often considered at the forefront of mental health advocacy, awareness, and investment. There has been significant government investment and expenditure to improve mental health care in Australia, with spending on mental health-related services increasing from $10.9 billion in 2017–2018, to $12.2 billion in 2021–2022 [7]. Yet the mental Health of Australians remains a major public health concern, with 43% of Australians experiencing a mental disorder at some point in their lifetime, and 22% experiencing disorder in the last 12 months [8]. The picture for younger Australians is more concerning, with 16 to 24 year olds more likely to experience high levels of psychological distress compared to older Australians, a trend that has been reported across numerous surveys and pre-dates the effects of the COVID-19 pandemic [4, 5, 12].

Australia’s first National Mental Health Strategy [6] pushed for improved accountability and integration of mental health services. Since its publication, decades of inquiries and reports have called for change to improve both the architecture of the mental health system, and the experiences and mental health outcomes of the Australians who are navigating it [23, 36]. Successive governments and decision-makers have grappled with the challenge of deciding what policies, programs, or set of actions should be implemented to achieve impact on mental health outcomes of Australians, within the constraints of finite resources and funding.

### Participatory systems modelling to support mental health decision-making

The role that empirical evidence plays in the decision-making process for mental health planning and funding is varied. The static nature of published health research may not always reflect the complex and dynamic needs of the relevant area of health [18, 47], and decision-makers can be affected by a range of political motivations and influences [10, 17]. Once mental health programs have been funded, ongoing evaluations of them are rare, limiting the ability of decision-makers to understand if they are achieving their intended impact and improving outcomes [16]. System dynamics modelling provides a compelling alternative to the decision-making ‘status quo’. System dynamics modelling refers to the computational method for simulating the behaviour of complex systems over time, allowing for systematic testing of various scenarios to examine a potential outcome [25, 53]. It has been utilised as a decision-support tool for decades across a range of sectors such as business, defence, engineering, and economics [25, 54]. More recently, systems modelling has increasingly been applied to the health sector, providing a virtual environment to identify which interventions, or combination of interventions will have the biggest impact on mental health and economic outcomes, guiding investment decisions [15, 44].

Whilst system dynamics modelling utilises academic literature and data sets to build and validate the model structure, participatory systems modelling (PSM) places additional emphasis on involving a range of stakeholders within the community to contribute their expertise and knowledge to the development of a decision-support tool, and engaging them in the collective learnings [20, 60, 61]. For clarity, we use the term ‘decision-support tool’ to refer to the final model and interface designed for use by decision-makers, and ‘PSM’ to describe the methodological process used to develop the decision-support tool. By enabling open communication and collaboration between stakeholders and systems modelling experts, the process of building the tool can be enriched through a transparent sharing of resources and knowledge. This could include the identification of gaps in academic literature or data, critiques of assumptions made within the decision-support tool, modifications to better capture the real-life characteristics of the local mental health care system, prioritisation and description of interventions to improve the mental health outcomes of young people, or other unique insights around how the decision-support tool could be used at a decision-making level [19, 20].

### Right care, first time, where you live program – research context

The *Right care, first time, where you live* program (henceforth referred to as ‘the Program’), led by researchers at The University of Sydney’s Brain and Mind Centre (BMC) in partnership with local Primary Health Networks, utilises PSM methods to build and deliver youth mental health decision-support tools to eight unique sites across Australia [20, 22]. The decision-support tool allows for exploration of the impact of different strategies to improve youth mental health outcomes, and incorporates a range of interventions (e.g., technology-enabled care, school—based suicide prevention programs, post-suicide attempt care, etc.), social determinant ‘what-if’ scenarios (e.g., family and domestic violence, unemployment, social cohesion, substance misuse, etc.), and the ability to scale up and down service capacity growth rates (e.g., general practice, psychiatric hospitalisation, specialist services, alcohol and other drug treatment services, etc.). The tool offers a range of projected outcomes to select from when simulating one of these strategies, or a combination of strategies (e.g., moderate to very high psychological distress, emergency department presentations, etc.). Academic output from the Program describes the decision-support tool in further detail [46]. The program aims to provide these tools to decision-makers at each site to 1) guide the allocation of mental health funds and resources, 2) understand the health and economic impacts of implementing various mental health interventions or combinations of interventions, and 3) support cases for further investment decisions that address the unique needs of each community. The PSM process to build the decision-support tool for each site utilises a series of three workshops, where a range of local stakeholders engage and contribute to the development of the tool through a series of group activities that aim to define and refine the model. The methods section provides further detail on the broad objectives of each workshop, and the associated activities.

The Program pioneers a more organic, inclusive, and flexible PSM workshop structure than what has been traditionally utilised in PSM, which historically has relied on ‘scripts’ to guide workshop activities with smaller groups (20–25) [1, 26]. The current program forgoes the use of pre-determined scripts, and includes a larger group of stakeholders (50–70) to provide opportunities to collect a broader and unconstrained set of nuanced, authentic, and open stories and experiences from stakeholders. Further details on these PSM workshop methods, and the resulting decision-support tool have been published prior [20, 45, 56].

### The role of stakeholder engagement

The practical application of PSM to design and implement decision-support tools to inform mental health policy and planning however, remains a challenge, and is less understood [3]. The mobilisation of knowledge to inform health policy and planning is often highly context dependent and can vary depending on the local implementation environment [21, 62]. What has been understood, however, is that genuine stakeholder engagement is an important contributing factor in supporting the implementation of mental health projects into complex environments, and in the mobilisation of knowledge into the ‘real world’ [22, 39, 52, 58, 59]. To effectively embed stakeholder engagement to support this process, it is crucial to understand the specific methods and processes within PSM workshops that promote and foster stakeholder engagement.

The value of partnering with stakeholders via approaches such as ‘participatory action research’ or ‘community-based participatory research’—two methods emphasising an equal contribution of academics and community members to the research and implementation process—has been well-documented, including within the youth mental health space [24, 49, 62, 63]. The practical methods and processes that can facilitate stakeholder engagement and effective group contributions in participatory research have also been explored and evaluated in health [27, 28, 30, 37]. However, the application of a participatory-based research approach within systems modelling for youth mental health is relatively new, and evaluations of this process are more limited [32, 33]. Considering the impact that differing contexts have on the value and outcomes of PSM, evaluation is key in understanding the factors that drive stakeholder engagement in PSM within the youth mental health context. Given the current Program’s adoption of a more novel and adaptable approach to PSM workshops [20], there is a need to examine how this approach may enhance stakeholder engagement and knowledge sharing, as informed by participants’ perspectives and experiences of the workshop.

### Objectives

This paper aims to examine and document the explicit processes, interactive activities, and dynamics of the PSM workshops that seek to facilitate strong and genuine stakeholder engagement, and the sharing of stakeholder knowledge and experiences of the local mental health system. More broadly, this paper aims to examine the more flexible and adaptive approach to running PSM workshops as part of this Program, and how this approach to workshop facilitation can enable valuable stakeholder contributions to the development of the decision-support tool.

## Methods

### Research context and study site

The current study draws from an evaluation framework that has been established for the Program, which aims to understand the feasibility, value, impact (change and action), and sustainability of PSM processes for youth mental health within this Program [32]. This article applies this evaluation framework to one of the eight participating sites within this Program: Brisbane South Primary Health Network (PHN), located in Queensland, Australia. The Brisbane South PHN is an independent, not-for-profit organisation funded by the Australian Government. The PHN works to commission new health services and align and connect existing health services, to directly respond to the health care needs of the Brisbane South region.

### Participants and recruitment

The terms *participants* and *stakeholders* are both used in this paper. By *stakeholders,* we refer to the individuals who were invited to, and attended the PSM workshops, and contributed their knowledge, expertise, and experiences to the development of the decision-support tool. By using the term *participants,* we are referring to the stakeholders who consented to and completed an interview for the current evaluation of the PSM processes.

**Fig. 1:**
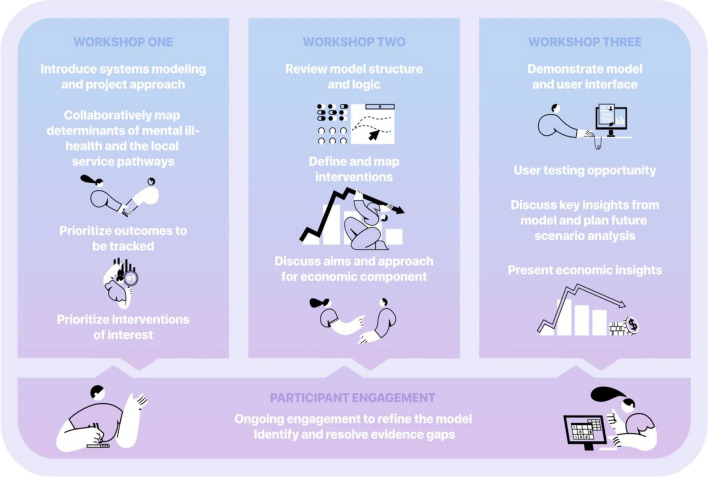
Overview of structure and content of workshops 1–3. Figure reproduced from [20] “Participatory Methods for Systems Modeling of Youth Mental Health: Implementation Protocol”, JMIR Mental Healt, Under CC BY 4.0* (* 10.2196/32988*)*

A wide range of stakeholders, representing diverse sectors of the Brisbane South youth mental health system (e.g., mental health professionals, service managers, health administrators) and the broader Brisbane South community (e.g., Queensland Department of Education, Youth Justice, Police), were invited to participate in the series of three PSM workshops which took place between March and October, 2023. The workshops included both informative presentations on the modelling process, as well as interactive group activities such as systems mapping exercises, facilitated group discussions, and opportunities to user test and provide feedback on the decision-support tool once built. Fig. 1 provides an overview of the three workshops’ structure and content.

The recruitment of stakeholders for the three workshops was governed by the participating site, Brisbane South PHN, who utilised their knowledge and partnerships in the region to identify and invite key stakeholders to attend the workshops via email. Further snowball sampling was utilised to invite additional stakeholders, to ensure diverse community members were represented. Stakeholders who attended at least one PSM workshop were invited by The University of Sydney’s BMC research team to participate in the current evaluation study via email. Informed consent was collected via the Participant Information and Consent Form, returned by participants to researchers via email.

### Procedure and study design

Qualitative evaluation data was collected via semi-structured interviews with participants at three timepoints: 1) at baseline, prior to the first workshop; 2) at follow-up timepoint 1 (T1), immediately following the final third workshop (i.e., six months after baseline); and 3) at follow-up timepoint 2 (T2) (approximately 12 months after baseline). All interviews were conducted by three members of the research team (S.P., V.L., G.Y.L) via online video or teleconference (e.g., Teams or Zoom). Semi-structured interviews, which took between 30 min and one hour, allowed for an in-depth understanding of participants’ thoughts and experiences of the Program, as well as for the collection of more contextualised data that conveyed a greater understanding of the region. The interviews captured four main factors: 1) the current state of the mental health system in the Brisbane South region; 2) the motivations, expectations and experiences of participating in the PSM workshops; 3) connections and collaborations with other organisations or people within the Brisbane South mental health community; and 4) experiences of using the decision-support tool. This paper focuses on 2) the motivations, expectations and experiences of participating in the PSM workshops.

### Data analysis

Semi-structured interviews were recorded via Microsoft Teams and transcribed into text via Otter.ai, an online tool engineered to transcribe audio or video speech directly to text [48]. Transcriptions were re-read for quality assurance by members of the research team, de-identified, and stored on the secure password-protected Research Data Store provided by [56] Transcriptions were uploaded to NVivo, a software program designed to support the analysis of qualitative data [43]. Thematic analysis of the data was undertaken, with three qualitative research team members (S.P., V.L., G.Y.L.) independently coding the interviews, and iteratively developing independent codebooks (Braun & Clarke, 2008). Fortnightly meetings were established between the research team members to discuss codes, context, and interpretations of the data, and to establish consensus on a final book of codes.

A subsequent analysis of codes, and development and identification of key themes was undertaken by a member of the research team (S.P.). This process was guided by the overarching research question which aimed to understand what methods, processes, or dynamics within the PSM workshops best enabled stakeholder knowledge sharing, and authentic stakeholder engagement. The goal of understanding what workshop elements facilitate the optimal environment for stakeholders to share their knowledge and expertise, and genuinely engage them in the workshops and the Program more broadly, was used as a lense to analyse the recurring patterns, overlaps, and relationships between codes, to inform the final themes. Eleven sub-themes were developed, and subsequently grouped into three broader themes: 1) diverse stakeholder representation; 2) diverse facilitation and data collection methods in the workshops; and 3) engaging communication and education around the Program. Original transcripts of interviews were regularly re-visited and read, and final themes were discussed with additional members of the research team (O.I., V. L.) to assess whether the themes were comprehensive and representative of the data.

## Results

### Demographics of participants

A total of 37 participants consented to, and participated in a baseline interview, 17 participated in follow-up timepoint 1 (T1), and 18 participated in the second follow-up timepoint (T2). A total of eight participants took part in both a baseline interview and the first follow-up interview, and four participants took part in all three timepoints. Participants’ professions are displayed in Table 1.

**Table 1:** Participant information

	Baseline		Follow-Up T1		Follow-Up T2	
	n		n		n	
**Total completed interview**	37		17		18	
**Profession/role**	**n**	**%**	**n**	**%**	**n**	**%**
Health manager/administrator	20	54	10	63	12	66
Clinician/front-line health worker	14	38	5	31	3	17
Young person with lived experience	3	8	1	6	3	17

The following results outline the key themes that emerged from the analysis of interview data.

#### 1) Diversity in stakeholder representation

Ensuring a diverse representation of stakeholders at the workshops was cited by participants as a crucial element in ensuring that the information collected from stakeholders accurately reflected the local community and mental health system. Stakeholder diversity also played a role in creating a supportive environment where all groups of stakeholders felt comfortable contributing to the workshop activities. Numerous participants emphasised that the Brisbane South region is not only home to a diverse population, with a rich blend of cultural backgrounds and languages, but it is also geographically diverse, encompassing urban, regional, and more remote areas, as well as a significant variation in services that cater to these diverse regions. One participant noted, “*Because Brisbane South PHN geographically is a very large land area… I do think some of those more remote areas did get captured… so I think there was a fairly good geographic representation in the room*” *(Health manager, T1)*. Therefore, diversity in stakeholder representation emerged as a key component in the workshops, and extended not only to individual stakeholder background and the inclusion of stakeholders with lived experience, but also to diversity in sector representation, and professional and non- professional stakeholders, as outlined below.

##### Diversity in stakeholder background

Diversity in the personal background of stakeholders was expressed by participants as an important element to include in PSM workshops. This included diversity in stakeholder age, gender, sexual orientation, or cultural identity. While a participant acknowledged the “amazing attempt” to include as many voices as possible (*Mental health professional, Baseline*), there was variance amongst participants as to how successful the workshops were at including this diversity:

“For me, it’s how do we start to bring more diverse voices to the table? First Nations and LGBTI communities… more young people’s voices” (Health administrator, T1).

However, participants also acknowledged the challenges in recruiting stakeholders representing minority groups or services within the community (e.g., LGBTQIA+ individuals, transcultural mental health services), especially to a process that may have been perceived as academic (i.e., the academic aspect of this Program may have presented as intimidating or alienating to young people). This sentiment was expressed by a participant representing a culturally and linguistically diverse service, who noted a significant gap in the representation of services that cater to individuals with diverse cultural backgrounds. They noted, “*if you want [those services] there, then you need to make the invitation a bit personal*” *(Mental health professional, T1)*, highlighting that the engagement of stakeholders that deliver culturally-informed care may benefit from more targeted and personalised engagement, early in the workshop recruitment process. That is, ensuring the invitation conveys a clear understanding from researchers of some of the unique challenges of this area of work (e.g., limited funding and specialised services for migrant and refugee populations, unique cultural needs of their clients), and that their contributions have value.

Further, a larger representation of stakeholders from a minority group or minority service was flagged as an important factor in supporting these stakeholders to feel confident in contributing their knowledge to the workshop. One participant noted that in workshops one and two it was easier for them to contribute as a transcultural mental health professional, given they felt support from the handful of other transcultural health workers in the room. In workshop three, when these stakeholders were not present, the participant expressed that it was, “*a bit awkward to take the conversation from the whole group. Even if I bring something up, then there’s no one else there to support me or to add to the conversation” (Mental health professional, T1)*. The participant described this experience *as “tiring”*.

##### Inclusion of young people with lived experience

The inclusion of young people with lived experience was widely considered to be crucial in understanding the experience of those utilising the mental health system, as one participant noted, *“we so often only hear from the ‘experts’” (Mental health service manager, T1).* There was general consensus from participants that there needed to be greater representation from young people at the workshops. The challenges of navigating the mental health system were widely acknowledged by participants, yet there was a lack of representation from young people to speak to their experience. Also crucial in understanding this multidimensional picture of lived experience was the inclusion of young peoples’ support people (e.g., family member, friend, carer), with an acknowledgement that sometimes the support person is the one “*to help that young person navigate that mental health system”,* and that they are a *“key stakeholder”* (*Mental health service manager, T1*).

Regarding the young people who did attend the workshops, one participant stated that they felt that young people were *“valued, acknowledged and given the space”* to contribute in the workshops *(Mental health service manager, T1)*. However, several young people reported that in the first workshop that there was an element of “*infantilization”,* and that “*[It felt] like we weren’t there of value. We were there kind of tokenistically, the way we were being spoken to by other service providers” (Young person, T2).* It was noted by young people that by the time they attended the second and third workshops, this dynamic had improved, and that there was* “more respect” (Young person, T2).*

##### Diversity in sector representation

Participants expressed the importance of a wide representation of health and social sectors at the workshops. Diverse sector representation should include State and Nationally-funded public services and service providers (e.g. *headspace* centres, child and adolescent mental health services, Metro South Health, etc), Non-Government Organisations/Non-Profit Organisations (e.g., Wesley Mission Queensland), and the private sector (e.g., private psychology clinics, private hospitals). The importance of this diverse sector representation emerged as particularly important for participants considering how divided these sectors are, and the limited collaboration between them (e.g., due to funding structures, the cohorts they deliver care to, bureaucratic divisions, etc). Therefore, excluding one or more sectors could discount input from a vital component of the region’s mental health system, and the role it plays within it. For example, one participant noted how the private sector can be overlooked, given they are not awarded structured “*funding or grants or contracts*”. They stated, “*They’ll often be overlooked…but they’re definitely doing community-based work to keep these young people out of hospital*” (*Mental health professional and health administrator, T1*). Participants also valued the representation from sectors that *“sit slightly on the fringe, not strictly the mental health sector”, (Health administrator, T1)* acknowledging the value and relevance of areas such as youth justice, education or child protection.

##### Balance of professional and non-professional stakeholders

Many participants expressed the importance of maintaining an equal balance of health experts working professionally in the field (e.g., health managers and administrators, mental health professionals) and non-professional community members (e.g., young people, individuals with lived experience of mental ill-health, as well as their carers or support people). One participant expressed a concern that the workshop was “*fairly top-heavy*” *(Mental health professional, T1).*

“*Yes, we had diverse representation… [but] there was a lot of management in the room”, (Health administrator, T1).*

Ensuring an equal balance of power was expressed as important not only *“to try and get… different perspectives”* (*Mental health professional, T1*), but also to ensure the conversation remained balanced (i.e., avoiding tangents), and to ensure all voices felt comfortable in speaking up. One young person with lived experience described speaking in an environment with health professionals as *“a bit intimidating”,* however, having other young people at the table created a “*safe space”* for them *(Young person, T1)*.

Conversely, ensuring that a level of seniority was represented at the workshop was cited as a factor that could not only provide insight at the workshops from a decision-making level of health, but could potentially influence the trajectory of the program moving forward, in terms of translation and implementation of the decision-support tool. One participant noted, “*effective partnerships and effective working together often relies on having the right people in the right position at the right time*” *(Health administrator, T1).*

Despite broad support from participants regarding diversity of stakeholder and sector representation, some participants acknowledged the substantive time and resources involved for stakeholders in participating in the workshops, with one participant expressing that the inclusion of more representation was not always of additional value to the PSM process. That is, while diverse representation of groups was crucial, they felt that larger numbers of stakeholders within that represented group was not necessarily required.

*“More wasn’t necessarily better, I think, and I think that’s what I’m sort of leaning towards in terms of the investment of resources versus the outcome. It wasn’t just a case of, the more diversity, the more representation we can get, the better outcome is going to be. Actually, I’m not sure about that. I think the same outcome could have been achieved with a smaller group” (Health administrator, T2)*.

#### 2) Multiple data collection methods

The PSM workshops utilised a range of facilitation methods to effectively capture rich stakeholder feedback. There was consensus from participants that flexibility in facilitation methods, and the provision of multiple feedback channels for stakeholders, ensured that all stakeholders had adequate opportunity to contribute to the workshops. Participants noted the value of having feedback options (including an anonymous option), particularly for stakeholders who may have experienced barriers in providing their contributions, as outlined below. The themes below indicate factors in the workshops that facilitated the collection of diverse feedback.

##### Active facilitation of group activities

Many participants expressed that the group activities that were conducted around large tables and encouraged group conversation, could sometimes be monopolised by a handful of louder, more confident stakeholders, limiting the contributions of others. One participant noted, *“in large groups like that it’s sometimes hard… everyone has their own focus a lot of the time” (Mental health service manager, T1).* They noted one instance where some stakeholders *“sort of sat back and gave up a little bit”* (T1). Active and directive facilitation from the research team facilitators allowed the management of the group conversation, including the monitoring of time, guiding tangential topics of discussion when required, moving on from a topic once saturated, and importantly, prompting stakeholders to ensure they had an opportunity to speak, (e.g., younger stakeholders) – *“I think that facilitator did as much as they possibly could” (Service manager, T1).*

##### ‘Roving’ facilitators

Participants expressed the importance of the research team capturing more informal and conversational feedback that occurred during group activities, by providing a ‘roving’ facilitator to take notes of more ‘ad hoc’ conversations that occurred during the group activities. These facilitators differed from a tradition group facilitator in that they moved between stakeholder groups, prompting and listening to conversations with stakeholders who may not have been contributing as much feedback or who were not well-heard. One participant expressed, *“I think that that was really good to have those facilitators there, for someone to sort of move around and maybe connect more with certain people that…felt like they had a lot to say” (Service Manager, T1).*

##### Anonymous feedback option

A QR code provided by the research team allowed participants an option of providing feedback confidentially and anonymously, via an online form, and provided an important alternative to contributing feedback when *“not everyone feels comfortable speaking up” (Service manager, T1)*. Barriers reported by some participants that may have prevented them from contributing their knowledge and experiences in the workshop activities included other competing voices, and the impact of power dynamics within the group. For example, one young participant described speaking up within the group context as *“quite overwhelming”,* and that they didn’t feel *“good on the spot”* (referring to answering questions directly) *(Young person, T1).* An option to provide feedback anonymously ensured the potential for contributions from all stakeholders irrespective of their confidence levels, political affiliations within the room, or professional seniority.

##### Workshop environment

Participants communicated that the workshop environment impacted their ability to actively contribute to the workshop. Participants cited a number of logistical practicalities to support contributions, such as a large room to maximise space for stakeholders to interact and allow sufficient space to separate large group activities and their associated noise, and to include ‘break-out’ areas for those with lived experience to access if they became overwhelmed. The option of workshop facilitators actively grouping participants together was also raised, with one young participant suggesting that facilitators could *“put some young people together” (Young person, T1)* in the group activities to help them feel more comfortable and confident in contributing. Conversely, the majority of participants saw value in including a range of diverse stakeholders within one conversation. Consistent with the aims of a participatory research approach, this finding highlights the importance of stakeholders from multiple perspectives contributing together and challenging each other, however, it also spotlights the need for potential additional consultation with young people separately to ensure their input is adequately captured.

#### 3) Education and communication

Clear and engaging communication and education around the Program emerged as an important theme in empowering stakeholders to contribute to and engage with the workshops. Embedding this education into the workshop, and including the aims of the Program, and why the stakeholders were invited to be a part of it, was cited by participants as a critical component in driving their engagement with the workshops and the program more broadly.

##### Empowering stakeholders through education

Ensuring stakeholders understood the scope and aims of the Program, and how it differentiated from other competing initiatives, was a key element in empowering them to meaningfully participate in the workshops. One participant acknowledged, prior to the first workshop, that *“our [mental health] system is very complex”,* and considering the various initiatives already being rolled out in the Brisbane South region, posed the question, *“where might [they] factor into the modelling… how do they align with it? Or are they separate?” (Health administrator, Baseline).* Another participant described themselves as *“very sceptical” (Health administrator, Baseline)* in the early stages of the program regarding the decision-support tool, and prior to attending the workshops. By the end of the final workshop, the participant described themselves as *“an ally” (T2)* to the program. One participant illustrated the importance of empowering stakeholders through knowledge, and ensuring they have the *“vision”.* They stated, *“it keeps going back to… people getting it. And you’ve got to take people along the journey” (Health administrator,T1).*

##### Managing the level of technical detail

A transparent approach was utilised in communicating and educating the stakeholders regarding more technical aspects of the PSM development process, and included information on sources of data utilised in the decision-support tool, as well as the literature and evidence that supports the tool’s interventions and their associated values. As discussed above, the approach of actively educating stakeholders proved beneficial, but conversely, many participants expressed that there was a limit on the need to understand the more detailed technicalities within the tool. One participant noted, “I imagine there is a massive, massive amount of information sitting in the back end of that model. I don't necessarily want or need to know all of that information… I think knowing that it's sitting there is useful, but I don't need to know the nuances,” (Health administrator, T1). Further, managing the right balance of information provided to stakeholders (i.e., limiting overly technical elements) allowed stakeholders to more easily engage with the content, and avoid what one participant described as not being able to “handle full transparency” (Health administrator, T1).

##### Increased understanding of other services

A common piece of participant feedback was that the workshops enabled a rare opportunity for interaction with stakeholders from a range of different services and organisations in the Brisbane South region, and that this experience had increased their education around these services and organisations, and challenged some pre-conceived views that they held. One participant expressed the value in better understanding the shared challenges and circumstances of others services, “because you don’t actually get to ever see this or understand this” (Mental health professional, T1). These additional learnings for stakeholders were a natural bi-product of participating in the workshops, and resulted in a strengthening of stakeholder engagement, and a motivation to attend future workshops.

## Discussion

PSM is increasingly recognised as a valuable participatory method that harnesses the local and historical expertise of stakeholders to bring crucial experiential knowledge to a model building process [34]. PSM has been distinguished from other participatory methodologies for its ability to surface ‘counter-intuitive’ insights in public policy situations characterised by conflict [50], with models serving as ‘boundary objects’ that promote collaboration between diverse communities [9]. Documenting the explicit processes, activities, and dynamics of PSM workshops that facilitate strong and genuine stakeholder engagement with the modelling process and the broader program allows for a greater appreciation of what it takes to enable the collection and sharing of rich descriptive knowledge from stakeholders about the complexities and consumer experiences of the youth mental health system in the Brisbane South region.

### Stakeholder recruitment considerations

The broad finding that diversity in stakeholder representation, including those with lived experience, was of value in PSM workshops has been acknowledged in prior research [13, 21, 24, 33, 38, 41, 64]. This finding is not unexpected, and aligns with a core goal of participatory research – in this case, to ensure that the community affected by the decision-support tool contributes to its development, with diverse input being crucial for accurate representation. Further, stakeholder engagement and input in the research process, particularly from young people with lived experience, has shown to increase the credibility and ‘real world’ uptake of youth mental health interventions, particularly when conducting research in discrete communities with unique needs, as the current Program is [2, 51, 62]. However, the current study lacked consensus among participants that stakeholder diversity was well-achieved in the workshops, highlighting the more complex considerations around cultivating ideal stakeholder representation. Some participants indicated that more stakeholder numbers to increase representation is not necessarily better, that a saturation point should be considered, and that there are inherent difficulties in recruiting more vulnerable stakeholders in the community.

These findings reflect a fundamental challenge in PSM, that is, the methods of identifying stakeholders, and the limited control researchers have in anticipating who will provide meaningful contributions to the PSM process [31]. For example, there are a range of factors that contribute to the final group of participating stakeholders, such as stakeholder motivations for participating [57], the unpredictable nature of snowball recruitment, and the reality that many young stakeholders with lived experience may struggle silently, and are outside of the care systems that may have facilitated their participation. Thus, stronger consideration is needed regarding the active recruitment methods that identify and engage stakeholders, such as earlier and more personalised recruitment strategies, and collaborations with organisations that can facilitate the identification of valuable stakeholders (e.g., youth/drop-in centres, services supporting marginalised or minority populations). A more targeted approach of stakeholder identification and recruitment may also help alleviate the ‘more is better’ default, and result in a more select group of stakeholders. Moreover, participant recruitment subject to external constraints or influences further reinforces the need for an adaptive and flexible approach to workshop facilitation.

Important to note is that the current Program’s workshop recruitment is governed by the partnering site, but there has been an increase in youth engagement in subsequent sites, supported by the research team. Whilst not detailed here, additional activities to support the recruitment and experiences of young stakeholders in the Program, commencing early in the Program timeline, have been undertaken. One important outcome of this additional youth engagement work in the Program has been the establishment of the National Youth Lived Experience Reference Group, which aims to provide youth lived experience expertise across all phases of the Program. A major output of this group has been the development of the Youth Lived Experience Framework, a highly generalisable ‘best practice’ guide to engaging and empowering young people to contribute to the design of mental health initiatives such as research, service design, education or resource development, or any project that involves young people as a key stakeholder [40]. Future work may consider evaluating the impact of these additional activities, including the Youth Lived Experience Framework, on the engagement and contributions of young stakeholders to the PSM process, particularly considering the research highlighting the importance of the relationship-building process in engaging stakeholders [14].

### Employing an adaptive PSM approach

Current findings support the use of flexible and adaptive workshop processes that meet the needs of the site (e.g., sites with high numbers of lived experience stakeholders etc.). Multiple data collection methods, such as active facilitation of group activities, ‘roving’ facilitators, and anonymous feedback options were vital in enabling ease of stakeholder contributions, adapting to stakeholder needs, ensuring balanced conversations, allowing multiple streams of feedback to be collected simultaneously to mitigate time constraints, amongst more. Whilst the effectiveness of adaptive strategies have been documented across participatory research for health more broadly [27], as well as how these strategies can support diverse or marginalised stakeholders in PSM across other industries [11], there has been limited application of this approach to PSM for youth mental health specifically. The current findings affirm the shift away from traditional, script-based group model building workshop practices that have been utilised by systems modellers, which prescribe a sequence of problem scenarios to be solved, and a pre-defined set of actions for stakeholders to undertake to solve them [1, 20, 22, 26]. Whilst this approach strives for similar diversity of stakeholder representation and input in the modelling process, it lacks the flexibility of workshop processes that are required to support knowledge-sharing and contributions of stakeholders. It is important to document these agile and adaptive workshop methods utilised in PSM within the youth mental health space, particularly considering the often complex and contrasting perspectives and experiences of stakeholders in this sector.

The importance of adaptive workshop activities was highlighted via participants’ feedback regarding a need for comparable representation of professional and non-professional stakeholders to manage any power imbalance between these groups in contributing to the workshop. Importantly, the Program inherently requires the active input of health professional stakeholders, and senior health management stakeholders (e.g., Brisbane South PHN staff) who also support the coordination of the workshops. Thus, limiting the number of ‘professional’ stakeholders can only be achieved to a certain point. However, this feedback reinforced the flexibility required to manage the impact of a power imbalance—particularly, the provision of feedback preferences (e.g., anonymous online feedback) to ensure equal opportunities to contribute, and more active facilitation of workshop activities to balance the contributions of professional and non-professional stakeholders. Participant feedback around the value of contributions from experienced, senior health professionals in the workshops, highlights a secondary benefit of their involvement—that is, to help establish future partnerships and sustainability of the decision-support tool [42]. The impact of the Program workshops on stakeholder partnerships and cooperation across multiple Program sites has been explored in further detail [35].

### Stakeholder engagement through education

Participants valued clear and engaging communication and education regarding the aims and value of the PSM process and program more broadly. Providing program education empowered stakeholders to confidently and meaningfully contribute to the workshops, and importantly, it increased their engagement in the PSM process. These findings are in line with broader community-based participatory research [29, 62], but also with existing PSM research, which reported that establishing trust with stakeholders, and fostering a sense of empowerment in stakeholders to contribute to the PSM process, are key foundations to the PSM process and its future sustainability [33]. Embedding relevant education into the workshops, and “*taking people along the journey*”, highlights how knowledge sharing, and understanding the broader vision of the program is a key element in driving stakeholder engagement. Further, this outcome suggests that stakeholder engagement is an active process, that continues throughout the course of the three workshops, and can help build strong foundations for future partnerships (Goodman & Thompson, 2017). A future focus of the Program could examine ongoing partnerships or collaborations between stakeholders that were initiated as part of contributing to the Program, and how these collaborations might support the sustainability of the decision-support tool.

### Limitations

The representation of young people with lived experience who participated in an interview was limited, yet was broadly consistent with the limited representation of young people attending the workshops. The majority of feedback around diverse representation did not come directly from young people or minority groups, but was observed by health professionals – future evaluation can consider more direct and focused engagement of young people in workshop recruitment processes, to achieve a higher number participating in evaluation interviews and voicing their experiences directly. Further, a significant reduction of participants was noted in follow-up interviews compared to baseline. While the authors acknowledge a natural attrition in research, future recruitment should focus additional effort on this follow-up timepoint, to avoid attrition bias.

Box 1 provides a concise summary of the current paper’s findings as discussed in the Results and Discussion section, intended to inform future PSM workshops in the youth mental health space.

**Figure:**
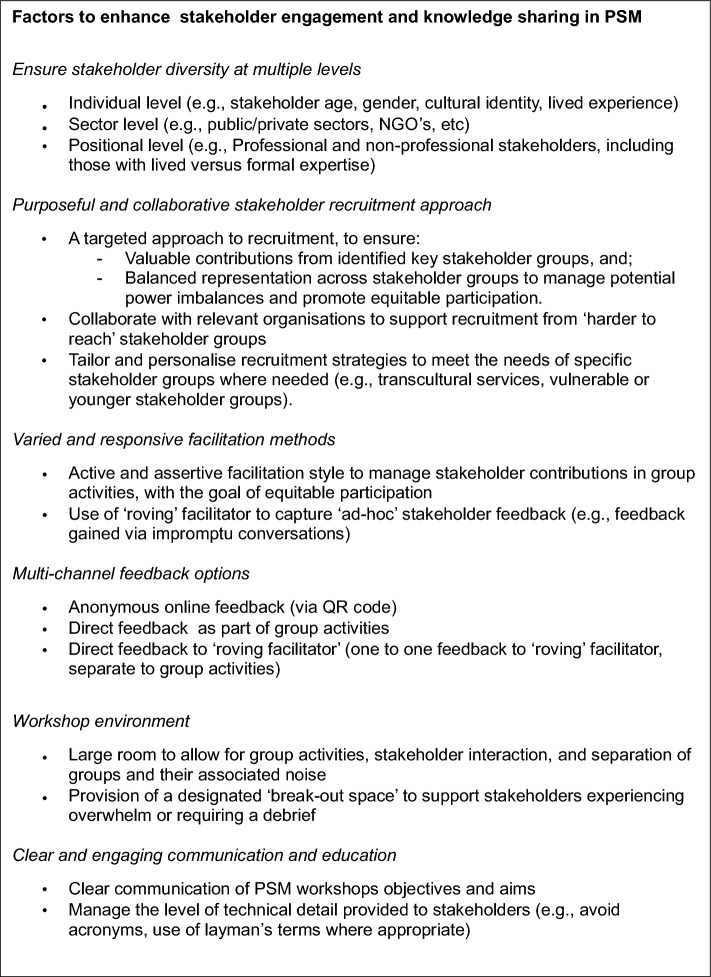
Box 1. Summary of key findings

## Conclusion

Participatory systems modelling workshops may not inherently guarantee strong stakeholder engagement and knowledge sharing, consequently, an adaptive and flexible approach to workshop activities that moves away from more structured workshop ‘scripts’, should be employed to engage diverse participants within the complex youth mental health space. Knowledge sharing and stakeholder engagement are dynamic, evolving processes cultivated throughout the course of the workshops. They are facilitated by education, and clear communication, which collectively empowers participants to contribute to the PSM process in meaningful and informed ways. Furthermore, the adaptive methods employed within these workshops warrant documentation and reflection, as such practices remain underreported in the PSM for youth mental health space. Capturing and sharing these approaches can guide future PSM in similar settings. Future PSM workshops should continue to develop additional activities and more targeted engagement with youth stakeholders to enhance their contributions.

## Data Availability

No datasets were generated or analysed during the current study.
